# Identification of Morphine and Heroin-Treatment in Mice Using Metabonomics

**DOI:** 10.3390/metabo11090607

**Published:** 2021-09-07

**Authors:** Wuhuan Lu, Ran Zhang, Wei Sheng, Luohua Feng, Peng Xu, Youmei Wang, Yuan Xie, Hui Xu, Guangji Wang, Jiye Aa

**Affiliations:** 1 Key Laboratory of Drug Metabolism and Pharmacokinetics, State Key Laboratory of Natural Medicines, China Pharmaceutical University, Nanjing 210009, China; 1822010331@stu.cpu.edu.cn (W.L.); ranzhang@stu.cpu.edu.cn (R.Z.); 3319010375@stu.cpu.edu.cn (W.S.); xuhui@cpu.edu.cn (H.X.); guangjiwang@hotmail.com (G.W.); jiyea@cpu.edu.cn (J.A.); 2 School of Pharmacy, China Pharmaceutical University, Nanjing 210009, China; 2020180686@stu.cpu.edu.cn; 3 China National Narcotics Control Commission—China Pharmaceutical University Joint Laboratory on Key Technologies of Narcotics Control, No. 24 Tongjiaxiang Road, Nanjing 210009, China; pengxu750@163.com

**Keywords:** heroin, morphine, serum and urine metabonomics, GC-MS, abuse

## Abstract

Although heroin and morphine are structural analogues and morphine is a metabolite of heroin, it is not known how the effect of each substance on metabolites in vivo differs. Heroin and morphine were administered to C57BL/6J mice in increasing doses from 2 to 25 and 3 to 9 mg kg^−1^ (twice a day, i.p.), respectively, for 20 days. The animals underwent withdrawal for 5 days and were readministered the drugs after 10 days. Serum and urine analytes were profiled using gas chromatography-mass spectrometry (GC-MS), and metabolic patterns were evaluated based on metabonomics data. Metabonomics data showed that heroin administration changed metabolic pattern, and heroin withdrawal did not quickly restore it to baseline levels. A relapse of heroin exposure changed metabolic pattern again. In contrast, although the administration of morphine changed metabolic pattern, whether from morphine withdrawal or relapse, metabolic pattern was similar to control levels. The analysis of metabolites showed that both heroin and morphine interfered with lipid metabolism, the tricarboxylic acid (TCA) cycle and amino acid metabolism. In addition, both heroin and morphine increased the levels of 3-hydroxybutyric acid and citric acid but decreased the serum levels of 2-ketoglutaric acid and tryptophan. Moreover, heroin and morphine reduced the levels of aconitic acid, cysteine, glycine, and oxalic acid in urine. The results show 3-Hydroxybutyric acid, tryptophan, citric acid and 2-ketoglutaric acid can be used as potential markers of opiate abuse in serum, while oxalic acid, aconitic acid, cysteine, and glycine can be used as potential markers in urine.

## 1. Introduction

The abuse of opiates has had an extremely negative impact worldwide and has become an increasingly serious social problem. Morphine is a prescription opiate, most commonly used for pain management, and can provide significant relief to patients suffering from pain [1]. However, prolonged use of morphine can lead to a situation of analgesic tolerance and dependence [2]. In contrast, heroin, or diacetylmorphine, is an opiate drug with two acetyl groups added to the basic structure of morphine [3]. Therefore, heroin is considered to be a prodrug, which can quickly enter the central nervous system through the blood-brain barrier and is finally hydrolyzed into morphine [4,5]. But heroin is a relatively common drug with high abuse potential and not approved for medical use in the United States [6]. Since 2010, not only has the rate of heroin-related drug overdose has risen sharply [7] but the number of deaths caused by heroin overdose has also increased more than fourfold [8,9].

Death from a heroin overdose invariably results from respiratory depression, and an overdose of opiates, such as morphine and fentanyl, can also cause respiratory depression [10,11]. In addition to respiratory depression, long-term use of opiates can cause clinical distress or impairment, commonly referred to as opiate-use disorder. Opiate-use disorders include dependence and addiction, and addiction represents the most serious form of the disorder [12]. In addition to changes in the basal nucleus and brain regions such as the nucleus accumbens [13,14], hippocampus [15,16] and amygdala [17,18], heroin or morphine addiction also involves reward [19,20], motivation [21,22], learning and memory [23,24] and changes the coupling among salience, default mode, and executive control networks [25,26]. However, heroin or morphine addiction is an uncontrolled, chronic, and recurrent encephalopathy that lacks specific and characteristic biomarkers for diagnosis and treatment.

The current study is designed to investigate the metabolic changes in the serum and urine after heroin or morphine exposure, withdrawal, and relapse, using an untargeted metabonomics method. Metabonomics, a global metabolic profiling method, offers insight into the mechanisms of opiate toxicity, and various biomarkers of opiate abuse can provide reliable forensic and clinical explanations [27]. Li et al. developed an ultra-performance liquid chromatography-time-of-flight mass spectrometry (UPLC-TOF/MS)-based metabolomic approach to evaluate the metabolic changes in the brain after chronic heroin exposure and withdrawal, and screened out candidate biomarkers of chronic heroin abuse [28]. However, due to the easy availability of blood or urine samples, candidate biomarkers in peripheral blood or urine are of more practical value than those in the brain.

In this study, we used a gas chromatography-mass spectrometry (GC-MS)-based metabonomics method coupled with multivariate statistical analysis to identify differentially expressed metabolites in the serum and urine of mice induced by repeated heroin or morphine exposure, withdrawal, and relapse. An important advantage of GC-MS is that it can identify a large number of substances in a single analysis of a very complex sample [29,30]. To further explore whether heroin and morphine have different effects on metabolism, the effects of morphine and heroin on endogenous metabolites were compared, and the potential biomarkers of opiate abuse were profiled.

## 2. Results

### 2.1. GC-MS Analysis of Serum

Representative GC-MS chromatograms of sera from the heroin-treated group and control group are shown in Figure S1, and typical GC-MS chromatograms of the sera from the morphine-treated group and control group are shown in Figure S2. At least 30 highly responsive metabolites were identified and assigned in the chromatograms of both heroin-treated and morphine-treated samples.

**Figure 1:**
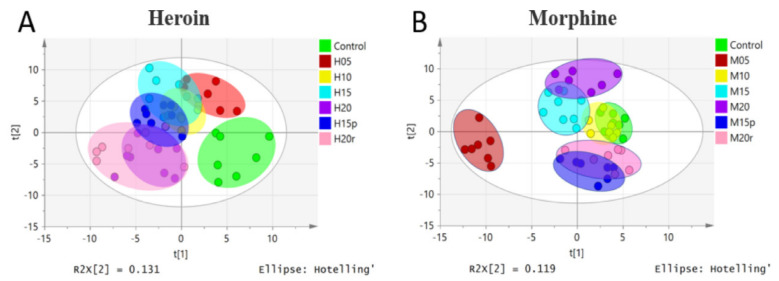
PLS-DA model of metabolic patterns for mouse serum. (**A**) Serum data from heroin-treated mice. Heroin was administered for 5 days (H05, red dots), 10 days (H10, yellow dots), 15 days (H15, light-blue dots), and 20 days (H20, purple dots). Mice were subjected to withdrawal (H15p, blue dots, withdrawal for 5 days after 10 days of heroin administration) and re-administration (H20r, pink dots); the control group is shown as green dots. (**B**) Serum data from morphine-treated mice. Morphine groups are labeled in the same way as heroin groups.

To obtain an overview of the data set, unsupervised principal component analysis (PCA) was applied. No outliers were found in the PCA model (data not shown). A partial-least-squares-discriminant-analysis (PLS-DA) loading plot is shown in Figure 1, and the score plot of heroin showed that samples from the same group clustered together, while samples from different groups were relatively scattered (Figure 1A). Samples from mice exposed to heroin for 5 days showed obvious deviation from the control value and continued to deviate after 10, 15, and 20 days of heroin administration, indicating heroin had an effect on serum metabonomics. The samples from mice 5 days after heroin withdrawal overlapped with those from mice exposed to heroin for 10 days, indicating that the disordered metabolism did not quickly return to baseline. However, samples from mice 5 days after heroin re-administration overlapped with samples from mice exposed to heroin for 20 days (Figure 1A); in other words, heroin re-administration changed the metabolomic profile again.

The PLS-DA loading plot of morphine-treated samples is shown in Figure 1B and displayed a different pattern than that of heroin. Specifically, the plots for mice exposed to morphine for 5 days deviated from the control plots significantly, but the plots for mice exposed to morphine for 10 days overlapped with those of the control plots, indicating that mice may develop resistance. After exposure to morphine for 15 and 20 days, the plots deviated from the control plot gradually. Interestingly, regardless of whether it was withdrawal or relapse, the plots were relatively similar to that of the control group.

### 2.2. GC-MS Analysis of Urine

Representative GC-MS chromatograms of urine from the heroin-treated group and control group are shown in Figure S3, and typical GC-MS chromatograms of urine from the morphine-treated group and control group are shown in Figure S4. At least 22 highly responsive metabolites were identified and assigned in the chromatograms of both heroin-treated and morphine-treated samples.

The PLS-DA loading plot of heroin in urine showed a pattern like that of heroin in serum (Figure S5A). In general, the data from the continuous heroin-administration group (H05, H10, H15 and H20) were clearly separated from those of the control group, and the data from the withdrawal group were slightly separated from those of the continuous heroin-administration group. The data from the relapse group were like the 20-day heroin-treated group. These findings indicated that heroin withdrawal was beneficial to the recovery of metabolic patterns to baseline.

The PLS-DA loading plot of morphine in urine also showed a pattern similar to that of morphine in serum (Figure S5B) but was not similar to that of heroin in urine. Briefly, the metabolic profiles of mice administered morphine for 5 days was significantly different from that of the control group, but that of the 10-day group overlapped with those of the control group; the metabolic profiles of the 15- and 20-day morphine groups deviated from those of the control gradually, and those of the withdrawal or relapse groups were similar to those of the control group. These findings revealed that mice may develop resistance to heroine with prolonged administration time.

### 2.3. Metabolic Effects of Heroin and Morphine Treatment on Serum Metabolites

**Figure 2:**
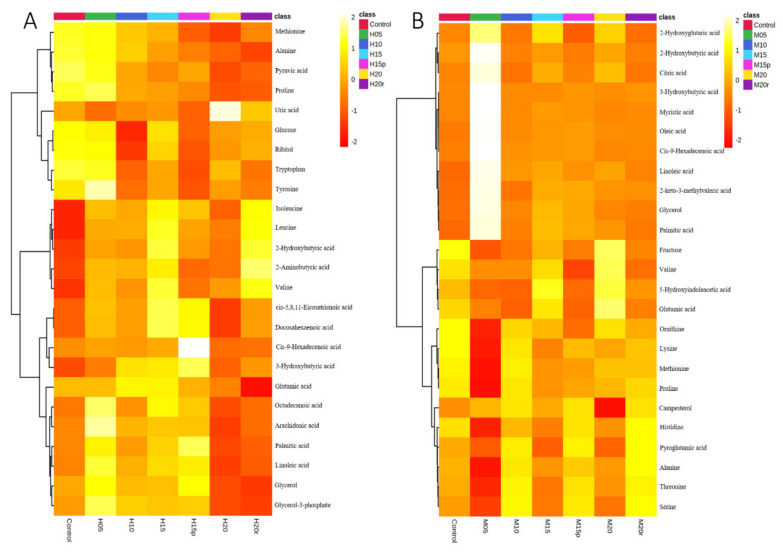
Heatmap of the serum metabolites from mice administered heroin (**A**) and morphine (**B**). Each row corresponds to a specific metabolite; each column corresponds to a set of samples (only group averages are shown). All the values were logarithmically transformed and are expressed as normalized values of the detected abundance of each metabolite. Red to orange indicates high to intermediate relative abundance, whereas orange to yellow indicates intermediate to low relative abundance.

The differential metabolites (TOP 25) in serum of seven groups are shown in a heat map (Figure 2). The heat map visually displays the change in metabolite abundance in different groups and is consistent with the PLS-DA loading plot for both morphine-treated and heroin-treated samples. As expected, the effects of morphine on metabolites in serum were somewhat dissimilar to those of heroin (Table 1). For example, the aspartic acid level increased after 15 days of continuous heroin administration but decreased after morphine administration for the same duration. These results suggested that, although heroin is considered a prodrug that can quickly reach the central nervous system and decompose into morphine, the metabolic regulations are not the same between heroin and morphine.

**Figure 3:**
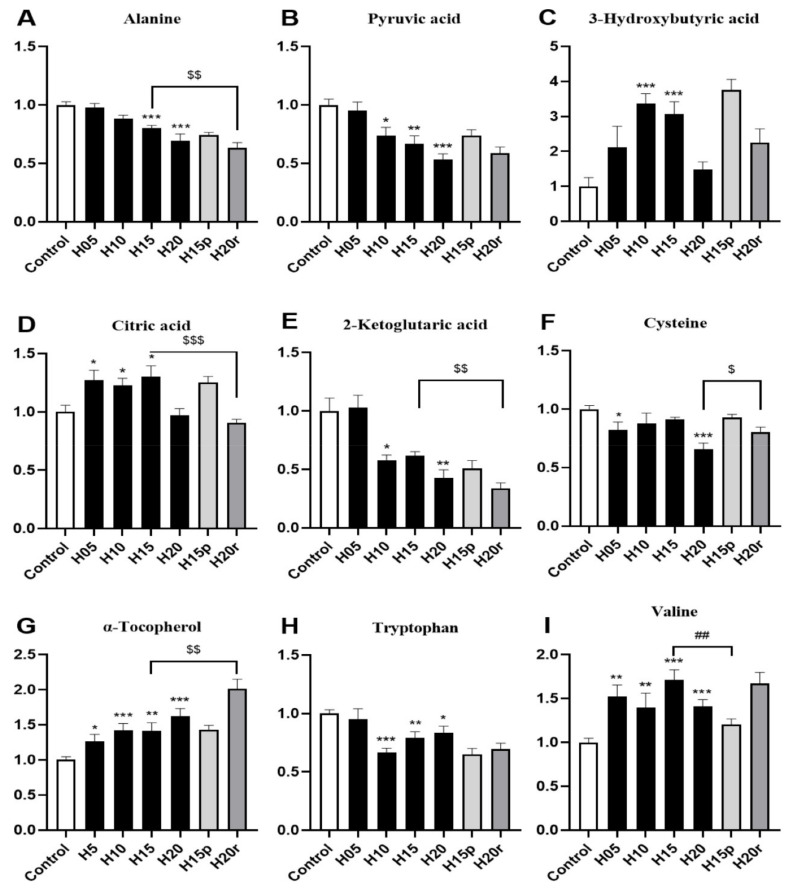
The effects of heroin on metabolites in serum. H5, exposure to heroin for 5 days; H10, exposure to heroin for 10 days; H15, exposure to heroin for 15 days; H20, exposure to heroin for 20 days; H15p, withdrawal for 5 days after 10 days of heroin administration; H20r, relapsing heroin use for 5 days (* *p* < 0.05, ** *p* < 0.01 and *** *p* < 0.001 compared with the control groups; H15p compared with H15, ^##^ *p* < 0.01; H20r compared with H15 or H20, ^$^ *p* < 0.05, ^$$^ *p* < 0.01 and ^$$$^ *p* < 0.001). (**A**) Alanine, (**B**) Pyruvic acid, (**C**) 3-Hydroxybutyric acid, (**D**) Citric acid, (**E**) 2-Ketoglitaric acid, (**F**) Cysteine, (**G**) α-Tocopherol, (**H**) Tryptophan, (**I**) Valine.

As shown in Figure 3A,B, the levels of alanine and pyruvic acid in serum decreased significantly after heroin administration for 20 days, which were metabolized by alanine aminotransferase (ALT) in the liver, suggesting this variation may be related to heroin-caused liver injury. In contrast, heroin resulted in a marked elevation in the 3-hydroxybutyric acid level, especially during the withdrawal period (Figure 3C), indicating that energy production may be activated by fatty acids. Interestingly, a decrease in the levels of citric acid and 2-ketoglutaric acid, two metabolites found within the tricarboxylic acid (TCA) cycle (Figure 3D,E), was observed, suggesting that heroin may affect cis-aconitase activity. In addition, heroin decreases cysteine and tryptophan levels and increases α-tocopherol and valine levels (Figure 3F–I), surprisingly, the tryptophan level did not return to the baseline level during heroin withdrawal. Instead, there was a decrease in tryptophan levels after withdrawal and an increase after relapse.

Morphine resulted in marked elevations in 3-hydroxybutyric acid, citric acid and α-tocopherol levels and reduced the levels of alanine, pyruvic acid, 2-ketoglutaic acid, cysteine, tryptophan and valine (Figure S6). Therefore, except for valine, the change in other metabolites in the morphine-treated serum were consistent with heroin. In summary, aspartic acid and valine can be used as potential markers to distinguish heroin from morphine in serum.

### 2.4. Metabolic Effects of Heroin and Morphine Treatment on Urine Metabolites

The differential metabolites (TOP 25) in the urine samples of seven groups are shown in a heat map (Figure S7). Similarly, the heat-map data was in accordance with the PLS-DA (Figure S5) data for both morphine-treated and heroin-treated samples. The effects of morphine on metabolites in urine were somewhat dissimilar to those of heroin (Table S1). For example, continuous injection of heroin for 15 days resulted in a decrease in palmitic acid, octadecanoic acid, and 3-hydroxybutyric acid levels, but these metabolites increased after 15 days of morphine injection. This result shows that heroin and morphine have different effects on the body.

**Figure 4:**
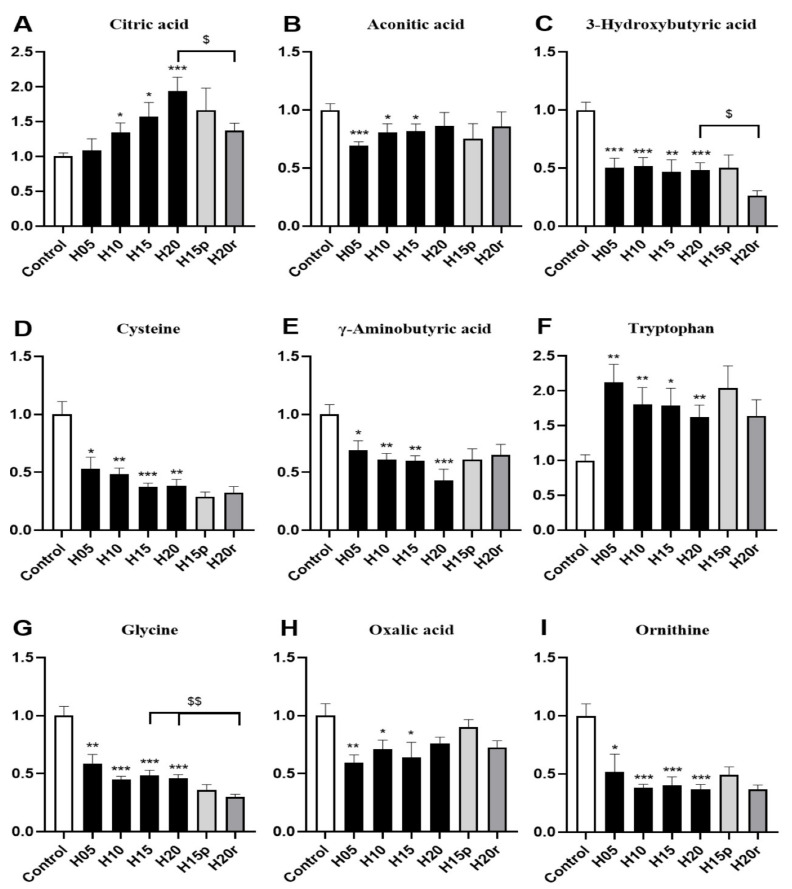
The effects of heroin on metabolites in urine. H5, exposure to heroin for 5 days; H10, exposure to heroin for 10 days; H15, exposure to heroin for 15 days; H20, exposure to heroin for 20 days; H15p, withdrawal for 5 days after 10 days of heroin administration; H20r, relapsing heroin use for 5 days (* *p* < 0.05, ** *p* < 0.01 and *** *p* < 0.001 compared with the control groups; H15p compared with H15; H20r compared with H15 or H20, ^$^ *p* < 0.05 and ^$$^ *p* < 0.01. (**A**) Citric acid, (**B**) Aconotic acid, (**C**) 3-Hydroxybutyric acid, (**D**) Cysteine, (**E**) γ-Aminobutyrix acid, (**F**) Tryptophan, (**G**) Glycine, (**H**) Oxalic acid, (**I**) Ornithine.

More concretely, the level of citric acid in urine gradually increased with the extension of heroin administration time (Figure 4A), and the level of aconitic acid in urine gradually returned to a normal level with increasing heroin administration time (Figure 4B), indicating that heroin interferes with the TCA cycle. Moreover, heroin administration can also cause the levels of 3-hydroxybutyrate, cysteine, gamma-aminobutyric acid, glycine, oxalic acid, and ornithine to decrease, especially after 20 days of administration (Figure 4C–I). It is worth noting that heroin exposure caused an increase in the level of tryptophan in the urine, but the level of tryptophan continued to rise after withdrawal and decreased after relapse (Figure 4F). Tryptophan levels were opposite in urine and serum samples of heroin-treated mice (Figure 3H), regardless of exposure, withdrawal, or relapse period.

Morphine significantly increased the levels of 3-hydroxybutyric acid, γ-aminobutyric acid, and ornithine and decreased the levels of citric acid, aconitic acid, cysteine, tryptophan, glycine, and oxalic acid (Figure S8). Hence, except for aconitic acid, cysteine, glycine, and oxalic acid, the change trend of other metabolites in morphine-treated mouse urine were different from those in heroin-treated mouse urine. Thus, citric acid, 3-hydroxybutyric acid, γ-aminobutyric acid, tryptophan valine and ornithine can be used as potential markers to distinguish heroin from morphine in urine.

### 2.5. Potential Markers of Opiate Abuse

Although heroin and morphine have different effects on the body, some endogenous metabolites exhibited similar changes and can be used as potential markers of opiate abuse. Both heroin and morphine exposure increased the serum levels of 3-hydroxybutyric acid (Figure 3C and Figure S6C) and citric acid (Figure 3D and Figure S6D) and decreased the serum levels of 2-ketoglutaric acid (Figure 3E and Figure S6E) and tryptophan (Figure 3H and Figure S6H). In addition, after withdrawal, the levels of 3-hydroxybutyric acid and tryptophan did not return to baseline levels and deviated more from normal levels than after administration, and this difference narrowed after relapse. In the TCA cycle, citric acid (which increased after heroin or morphine exposure) is converted into 2-ketoglutaric acid (which decreased after heroin or morphine exposure) via cis-aconitic acid and isocitrate dehydrogenase. Therefore, 3-hydroxybutyric acid, tryptophan, citric acid and 2-ketoglutaric acid can be used as potential markers of opiate abuse in serum.

Both heroin and morphine exposure reduced the urine levels of aconitic acid (Figure 4B and Figure S8B), cysteine (Figure 4D and Figure S8D), glycine (Figure 4G and Figure S8G) and oxalic acid (Figure 4H and Figure S8H). Interestingly, the level of oxalic acid decreased after the administration of morphine and heroin, recovered to normal levels after withdrawal, and decreased again after relapse. In conclusion, oxalic acid, aconitic acid, cysteine, and glycine can be used as potential markers of opiate abuse in urine.

## 3. Discussion

This study produced three main findings: (1) repeated heroin and morphine exposure caused significant metabolic changes in the serum and urine, and the differential metabolites mainly involved lipid metabolism, TCA cycle and amino acid metabolism; (2) after 5 days of morphine withdrawal intervention, most differential metabolites in the serum and urine were similar to control levels, but changes persisted in the serum and urine after heroin withdrawal; and (3) after 5 days of heroin relapse, the metabolites in serum and urine changed again, while some of the metabolites in serum and urine did not change significantly after 5 days of morphine relapse.

Specifically, the influences of heroin on serum and urine are not all the same, so too for morphine. For example, the changes of metabolites, such as 3-hydroxybutyric acid and tryptophan, in serum are opposite to those in urine, which can be used as potential biomarkers of heroin abuse, while aconitic acid, ornithine, and γ-aminobutyric acid in serum represent morphine. Compared with heroin, morphine significantly increased 3-hydroxybutyric acid, the levels of γ-aminobutyric acid and ornithine decreased the levels of citric acid. Therefore, citric acid, 3-hydroxybutyric acid, γ-aminobutyric acid, and ornithine can be used as potential markers to distinguish heroin and morphine in urine. This is the advantage of urine samples.

Lipids provide essential fatty acids for the body, and ketone bodies are intermediate products of the oxidative decomposition of fatty acids, including acetoacetic acid, β-hydroxybutyric acid (also known as 3-hydroxybutyric acid), and acetone [31]. Ketone bodies become an important energy source for the brain in situations where glucose is sparse [32]. A recent study indicated that heroin reinforcement results in impaired energy production through different pathways, including keto body metabolism, the TCA cycle and glycolysis. In addition, the significantly increased levels of 3-hydroxybutyric acid and acetoacetate in serum indicated that energy production from fatty acids was activated [33]. Despite the limited evidence in published studies, the data in this study strongly suggest that repeated heroin and morphine exposure altered energy metabolism (e.g., lipid metabolism and the TCA cycle). However, heroin (or morphine) affects not only energy metabolism but also amino acid metabolism. Some studies have shown that the level of tryptophan decreased significantly in the peripheral blood of morphine- and heroin-addicted rats, suggesting that addiction to morphine and heroin was related to tryptophan uptake from the blood by the brain [33,34,35]. The changes in tryptophan observed in this study may be related to drug addiction.

In addition, some studies have shown that 72.7% of aging, male Mexican-American long-term injection drug users (IDUs) with long-term heroin injection suffered from different forms of liver injury (such as hepatitis C, liver cirrhosis and hepatitis B) [36,37], and others have shown that heroin use is related to liver fibrosis [38]. It was generally believed that the liver injury of drug addicts was caused by sharing injection needles, while the liver damage effect of heroin itself was ignored. In our study, serum alanine and pyruvate significantly decreased after heroin administration, and they were metabolized by alanine aminotransferase (one of the indicators of liver injury), suggesting that heroin administration may cause liver injury.

This work not only focused on the long-term effects of opiates (heroin and morphine) but also explored the long-term effects after opiate withdrawal and relapse. Our data indicated that repeated heroin exposure induced a long-lasting metabolic effect in the serum and urine, whereas repeated morphine treatment induced a reversible effect in the serum and urine. This result may be due to the difference in resistance of mice to heroin and morphine. However, although the effect of withdrawal or relapse on metabolism is inconsistent between morphine and heroin, the serum tryptophan level of both morphine and heroin decreased further after withdrawal, while the level of tryptophan after relapse was similar to that before withdrawal. Tryptophan is the only precursor of serotonin, which is a key monoamine neurotransmitter involved in central nervous transmission and regulation of intestinal physiological function [39,40]. Thus, we speculate that the brain serotonin system may mediate some behavioral effects associated with heroin or morphine withdrawal. However, a recent study on the serotonin transporter (SERT) in the brains of individuals who use drugs showed that the SERT protein content did not change significantly [41]. The authors explained that their results were based on small sample size, were preliminary, and required replication. Moreover, it is necessary to study the SERT status of individuals who use drugs [41].

In summary, this study identified that the effect of heroin on the body was dissimilar to that of morphine. The endogenous metabolites with different changing trends can be used as potential markers to distinguish heroin use from morphine use, while the endogenous metabolites with the same changing trends can be used as potential markers of opiate abuse. However, both heroin and morphine exposure significantly interfered with the TCA cycle and lipid and amino acid metabolism. The change in tryptophan levels suggested that the brain serotonin system may mediate some behavioral effects of heroin and morphine withdrawal.

## 4. Material and Methods

### 4.1. Materials and Reagents

Reference standards of heroin and morphine were provided by the Jiangsu Institute for Food and Drug Control (Nanjing, Jiangsu, China). The internal standard (IS) compound myristic-1,2-^13^C_2_ acid (99 at.% ^13^C), alkane standard solution (C_8_–C_40_), methoxyamine hydrochloride (purity 98%), and pyridine (≥99.8% GC) were purchased from Sigma Aldrich. N-methyl-N-(trimethylsilyl)trifluoroacetamide with 1% trimethylchlorosilane were provided by Thermo Scientific (Bellefonte, PA, USA). HPLC-grade methanol and n-heptane were obtained from Tedia Company (Fairfield, CT, USA) and Merck (Darmstadt, Germany), respectively. Purified water was produced with a Milli-Q system (Millipore, Bedford, MA, USA).

### 4.2. Instrumentation

Chromatographic separation of the analytes was achieved using a Shimadzu GCMS-QP2010 (Shimadzu Corp., Tokyo, Japan) equipped with an RTx-5MS column (30 m × 0.25 mm i.d. fused-silica capillary column chemically bonded with a 0.25 μm cross bond, 5% diphenyl/95% dimethyl polysiloxane, Restek Corporation, PA, USA). A Sorvall Biofuge Stratos centrifuge (Sollentum, Germany) and an SPD2010-230 SpeedVac Concentrator (Thermo Savant, Holbrook, AZ, USA) were used to centrifuge the samples and evaporate the supernatants to dryness, respectively.

### 4.3. Animal Treatment and Sample Collection

Male C57BL/6J mice (6 weeks old, Vital River Laboratories, Beijing, China) were housed individually in metabolic cages with access to a standard chow diet under standard laboratory conditions with a 12 h light/dark cycle (lights were on from 7 AM to 7 PM) and temperature of 22 ± 2 °C. All experimental procedures involving the use of animals complied with the Guidelines for Animal Experimentation of the China Pharmaceutical University (Nanjing, China), and the protocol was approved by the Animal Ethics Committee of that institution.

After adaptation to standard laboratory conditions for 2 weeks, the mice were randomly allocated to four groups: the continuous administration group (heroin and morphine), the withdrawal group (heroin and morphine), the relapse group (heroin and morphine) and the control group, and the continuous administration group was further divided into 5, 10, 15, and 20 days of continuous administration (n ≥ 6 in each group). The heroin- and morphine-treated groups were administered intraperitoneal heroin or morphine in 0.9% saline, and the control was given an equal volume of vehicle. The mice were given heroin or morphine (or vehicle) twice daily for 20 days, with heroin administered at a dose of 2 mg kg^−1^ on the first day, increasing by 1.2 mg kg^−1^ d^−1^ to a final dose of 24.8 mg kg^−1^ and morphine administered at a dose of 3 mg kg^−1^ on the first day, increasing by 1 mg kg^−1^ every 3 days to a final dose of 9 mg kg^−1^. Heroin or morphine exposure was stopped for 5 days (the withdrawal period) after 10 days of administration, and then the mice were administered heroin or morphine again for another 5 days (the relapse period). Blood samples were collected on days 5, 10, 15 (exposure and withdrawal), and 20 (exposure and relapse) from the orbital venous plexus, and urine was collected 12 h before the blood samples were collected. The serum and urine samples were prepared and stored at 70 °C. All serum and urine samples were thawed at 37 °C for 20 min and mixed by vortexing before extraction.

### 4.4. Sample Preparation, Derivatization, and GC-MS Analysis

The serum and urine samples were pretreated, extracted, and derivatized as previously reported [42,43]. Briefly, an aliquot of serum (50 μL) was added to 200 μL of methanol with the IS myristic-1,2-^13^C_2_ (5 μg mL^−1^) and vigorously vortexed. An aliquot of urine (30 μL) was first added to urinase to decompose any excess urea and then added to 250 μL of methanol and vigorously vortexed. The supernatants of the serum (100 μL) and urine (60 μL) were then dried, methoxylated, trimethylsilylated, and analyzed via GC-MS [44]. To minimize systematic variations, all samples were analyzed randomly, and the quantitative data were normalized to the IS.

The peaks were automatically detected, the mass spectra were deconvoluted, and the GC TOF/MS data were acquired as reported previously [45]. Each of the endogenous compounds was identified by comparing the mass spectrum and retention index of the analyte with those of reference standards or those available in various libraries: mainlib and publib in the National Institute of Standards and Technology (NIST) library 2.0 (2008); Wiley 9 (Wiley-VCH Verlag GmbH & Co. KGaA, Weinheim, Germany). Compound abundance was quantified by calculating the area under the curve for the quantification ion of the compound.

### 4.5. Statistical Analysis

The relative quantitative data for the peaks (peak areas) were first normalized against the IS and then subjected to multivariate statistical analysis using SIMCA-P 13 software (Umetrics, Umeå, Sweden) [46]. To minimize the impact of the amount of urine from each rat, the urinary data were normalized to urine volume [47]. Principal component analysis (PCA) and partial least squares discriminant analysis (PLS-DA) were used to reduce the dimensionality of the data, and several principal components are used to describe the biggest difference between groups or samples. We took the two principal components t [1] and t [2] with the greatest difference as the two-dimensional diagram, t [1] as the abscissa and t [2] as the ordinate, while t [1] and t [2] of each sample point are different, such that they were scattered in the diagram and finally formed a scatter diagram. In this way, the PCA loading plot was obtained to observe the clustering, dispersion, and outliers of the sample, and the PLS-DA loading plot observed the difference between the groups. The results of the PCA or PLS-DA were interpreted as described previously [48]. Statistical analysis was carried out using SPSS software (Version13.0, SPSS). To determine the differences between two groups, independent sample T-tests were applied for the comparison of metabolite levels. Differences with a *p* value < 0.05 were considered statistically significant. Heat maps were generated using MetaboAnalyst [49].

## Figures and Tables

**Table 1:** Overview of the changes of serum metabolites about heroin and morphine.

Metabolic Pathways	Compounds	Heroin Serum Metabolites			Morphine Serum Metabolites		
		Exposure	Withdrawal	Relapse	Exposure	Withdrawal	Relapse
Lipid metabolism	Palmitic acid	↑↑	↑↑	↓↓	↑	-	-
	3-hydroxybutyric acid	↑↑	↑	↓	↑	↑↑	↑
	Cis-9-hexadecenoic acid	↑	↑↑	↓	↑	↑↑	-
Carbohydrate metabolism	Citric acid	↑↑	-	↓↓	-	↓	↓
	2-ketoglutaric acid	↓	↓	↓↓	↓↓	↑↑	↑
	Lactic acid	↓↓	-	↓↓	-	-	-
	Glucose	↓	↓↓	-	↓↓	↑↑	-
	Pyruvic acid	↓↓	↑	-	↓↓	↑↑	↑↑
GSH	Methionine	↓	-	-	↓↓	↑	↑↑
	Cysteine	↓	-	↓	↓↓	↑↑	↑↑
	Serine	↓↓	↑↑	↓	↓↓	↑↑	↑↑
Amino acid metabolism	Alanine	↓↓	↓	↓	↓↓	↑↑	↑↑
	Aspartic acid	↑↑	↓↓	↓↓	↓	↓	-
	Proline	↓↓	-	↓↓	↓↓	↑	↑↑
	Valine	↑↑	↓↓	-	↓	↓↓	↓
	Threonine	↓↓	↑	-	↓↓	↑↑	↑↑
	Tryptophan	↓↓	↓	↓	↓↓	↓	↓
	Isoleucine	↑↑	↓	-	↑	↓	↓
	Tyrosine	↓↓	↓↓	↓	↓↓	-	↓
Others	α-tocopherol	↑↑	-	↑↑	↑↑	↑↑	-
	5-hydroxytryptamine	↓	↑	-	↓↓	↑↑	↑

Note: the sign indicates the direction of change; ↓↓ for decrease, ↑↑ for increase, - for no change (*p* < 0.05, as indicated by the statistical analysis *t*-test); ↓ for decrease, ↑ for increase (there was a trend, but it was not statistically significant). Comparing the metabolites changes from the control group with the continuous heroin or morphine exposure group (15 days), and both the withdrawal group and the relapse group are compared with the exposure group for 15 days.

## Data Availability

The data that support the findings of this study are available upon request from corresponding author, Y.X., considering the complexity and large amount of data.

## Supplementary Materials

The following are available online at https://www.mdpi.com/article/10.3390/metabo11090607/s1, Figure S1: Representative GC/MS chromatograms of the sera from heroin-group and control, Figure S2: Representative GC/MS chromatograms of the sera from morphine-group and control, Figure S3: Representative GC/MS chromatograms of the urine from heroin-group and control, Figure S4: Representative GC/MS chromatograms of the urine from morphine-group and control, Figure S5: PLS-DA model of metabolic patterns for mouse urine, Figure S6: The effects of morphine on metabolites in serum, Figure S7: Heatmap of the urine metabolites after administration of heroin (A) and morphine (B), Figure S8: The effects of morphine on metabolites in urine, Table S1: Overview of the changes of urinary metabolites about heroin and morphine, Table S2: Overview of the changes of serum and urinary metabolites about heroin, Table S3: Overview of the changes of serum and urinary metabolites about morphine.
