# Supplementary material for: Identification of Morphine and Heroin-Treatment in Mice Using Metabonomics
Source: Metabolites. 2021 Sep 7;11(9):607. doi: 10.3390/metabo11090607 (PMC8467231; doi:10.3390/metabo11090607)
Supplement: Supplementary file 1 [file metabolites-11-00607-s001.zip › metabolites-1328315-supplementary.pdf]

Supplementary Material

Identification of Morphine and Heroin-Treatment in Mice Using Metabonomics

Wuhuan Lu [1], Ran Zhang [1], Wei Sheng [1], Luohua Feng [2], Peng Xu [3], Youmei Wang [3,*], Yuan Xie [1,*], Hui Xu [1], Guangji Wang [1] and Jiye Aa [1]

1 Key Laboratory of Drug Metabolism and Pharmacokinetics, State Key Laboratory of Natural Medicines, China Pharmaceutical University, Nanjing 210009, China; 1822010331@stu.cpu.edu.cn (W.L.); ranzhang@stu.cpu.edu.cn (R.Z.); 3319010375@stu.cpu.edu.cn (W.S.); xuhui@cpu.edu.cn (H.X.); guangjiwang@hotmail.com (G.W.); jiyea@cpu.edu.cn (J.A.)

2 School of Pharmacy, China Pharmaceutical University, Nanjing 210009, China; 2020180686@stu.cpu.edu.cn

3 China National Narcotics Control Commission—China Pharmaceutical University Joint Laboratory on Key Technologies of Narcotics Control, No. 24 Tongjiaxiang Road, Nanjing 210009, China; pengxu750@163.com

* Correspondence: and requests for materials should be addressed to youmei_626@163.com (Y.W.); yuanxie58@yahoo.com (Y.X.);

Figure S1. Representative GC/MS chromatograms of the sera from heroin-group and control,.

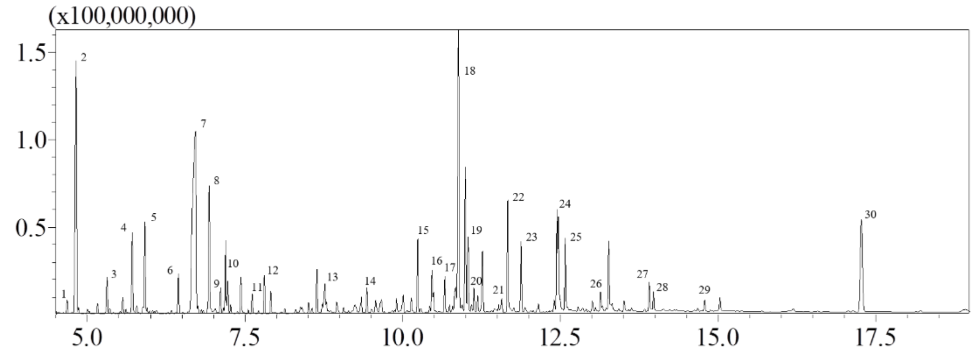

Note: 1. Pyruvic acid;2. Lactic acid;3. Alanine;4. Oxalic acid;5. 3-Hydroxybutyric acid;6. Valine;7. Urea;8. Phosphoric acid;9. Isoleucine;10. Glycine;11. Serine;12. Threonine;13. Pyroglutamic acid ;14. Phenylalanine;15. Glutamine;16. Citrate;17. Internal standard;18. Ribitol;19. Lysine;20. Tyrosine;21. cis-9-Hexadecenoic acid;22. Palmitic Acid;23. Myo-Inositol;24. Tryptophan;25. Octadecanoic acid;26. Arachidonic acid;27. cis-4,7,10,13,16,19-Docosahexaenoic acid;28. Monopalmitin;29. 1-Monostearin ;30. Cholesterol.

Figure S2. Representative GC/MS chromatograms of the sera from morphine-group and control,.

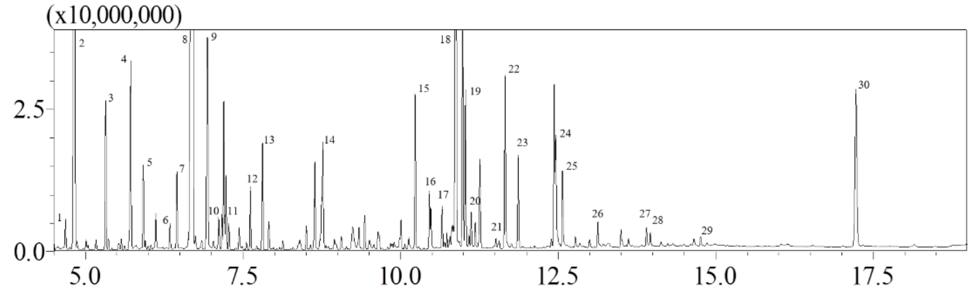

Note: 1. Pyruvic acid;2. Lactic acid;3. Alanine;4. Oxalic acid;5. 3-Hydroxybutyric acid;6. Acetamide;7. Valine;8. Urea;9. Phosphoric acid;10. Isoleucine;11. Glycine;12. Serine;13. Threonine;14. Pyroglutamic acid ;15. Glutamine;16. Citric acid;17. Internal standard;18. Ribitol;19. Lysine;20. Tyrosine;21. cis-9-Hexadecenoic acid;22. Palmitic Acid;23. Myo-Inositol;24. Tryptophan;25. Octadecanoic acid;26. Arachidonic acid;27. cis-4,7,10,13,16,19-Docosahexaenoic acid;28. Monopalmitin;29. 1-Monostearin ;30. Cholesterol.

Figure S3. Representative GC/MS chromatograms of the urine from heroin-group and control,.

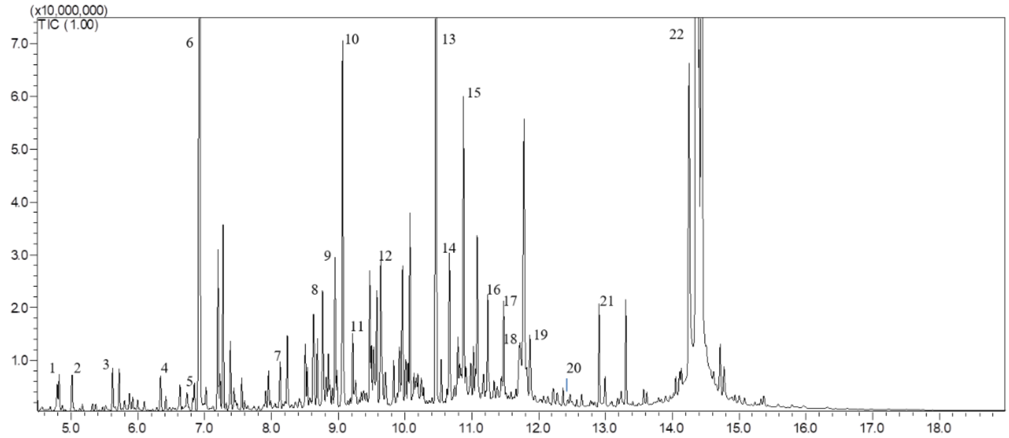

Note: 1. Lactic acid;2. Glycolic acid;3. Oxalic acid;4. Acetamide;5. Urea;6. Phosphoric acid;7. Beta-Alanine;8. Pyroglutamic acid ;9. Cysteine;10. 2-Ketoglutaric Acid ;11. Ornithine;12. Taurine;13. Citric acid;14. Internal standard;15. Ribitol;16. Glucitol;17. Beta-Methylglucopyranoside ; 18.Palmitic acid;19. Myo-Inositol;20. 5-Hydroxyindoleacetic Acid;21. Cystine;22. Lactose.

Figure S4. Representative GC/MS chromatograms of the urine from morphine-group and control,.

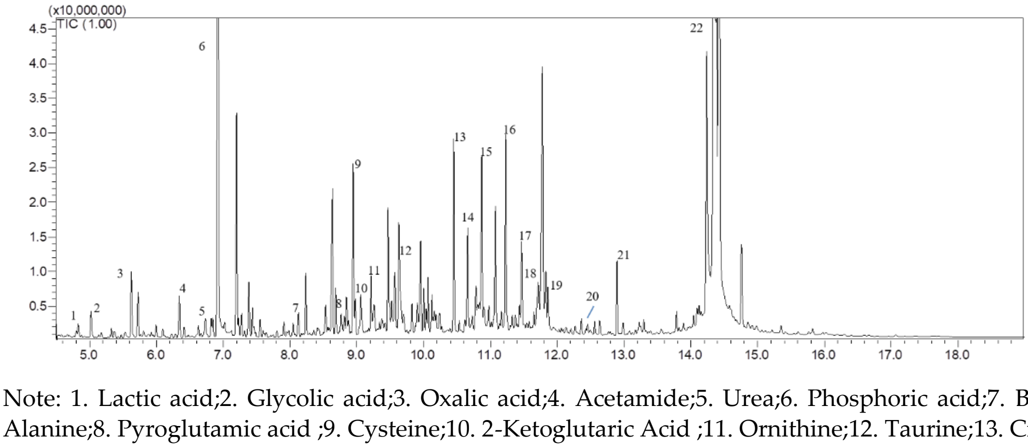

Note: 1. Lactic acid;2. Glycolic acid;3. Oxalic acid;4. Acetamide;5. Urea;6. Phosphoric acid;7. Beta-Alanine;8. Pyroglutamic acid ;9. Cysteine;10. 2-Ketoglutaric Acid ;11. Ornithine;12. Taurine;13. Citric acid;14. Internal standard;15. Ribitol;16. Glucitol;17. Beta-Methylglucopyranoside ; 18.Palmitic acid;19. Myo-Inositol;20. 5-Hydroxyindoleacetic Acid;21. Cystine;22. Lactose.

Figure S5. PLS-DA model of metabolic patterns for mouse urine,.

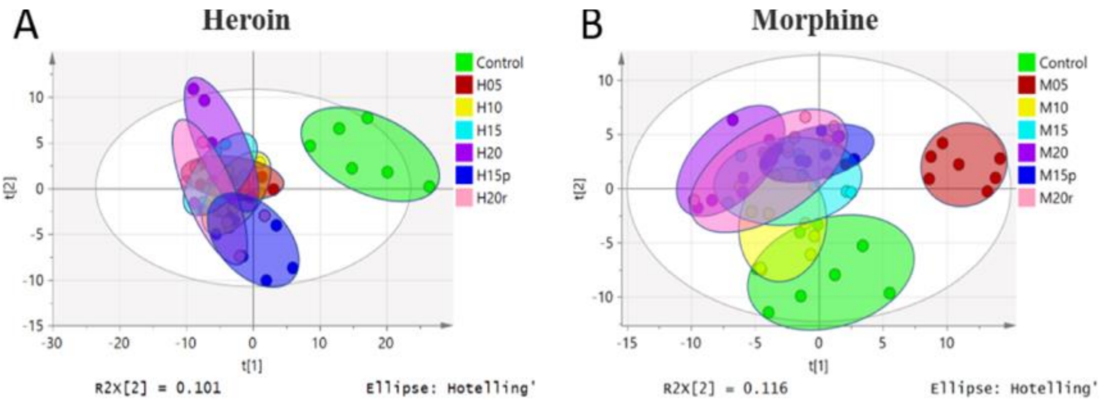

Note: (A) Urine data from heroin-treated mice. Heroin was administered for 5 days (H05, red dots), 10 days (H10, yellow dots), 15 days (H15, light-blue dots), and 20 days (H20, purple dots). Mice were subjected to withdrawal (H15p, blue dots, withdrawal for 5 days after 10 days of heroin administration) and re-administration (H20r, pink dots); the control group is shown in green dots. (B) Urine data from morphine-treated mice. Morphine groups are labeled in the same way as heroin groups.

Figure S6. The effects of morphine on metabolites in serum,.

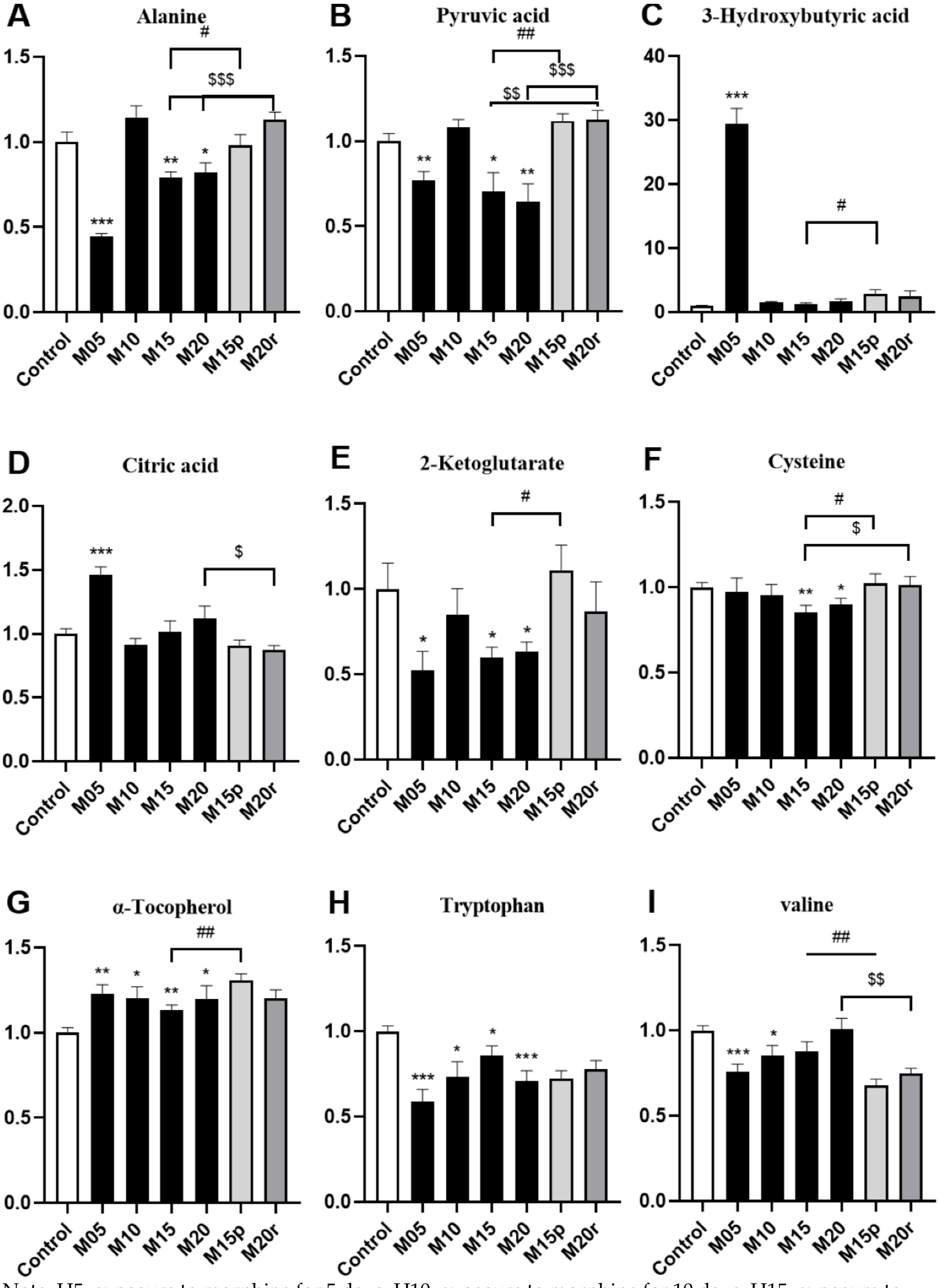

Note: H5, exposure to morphine for 5 days; H10, exposure to morphine for 10 days; H15, exposure to morphine for 15 days; H20, exposure to morphine for 20 days; H15p, withdrawal for 5 days after 10 days of morphine administration; H20r, relapsing morphine use for 5 days (*p < 0.05, **p < 0.01 and ***p < 0.001 compared with the control groups; H15p compared with H15, #p < 0.05, ##p < 0.01 and ###p < 0.001; H20r compared with H15 or H20, $p < 0.05, $$p < 0.01 and $$$p < 0.001).

Figure S7. Heatmap of the urine metabolites after administration of heroin (A) and morphine (B),.

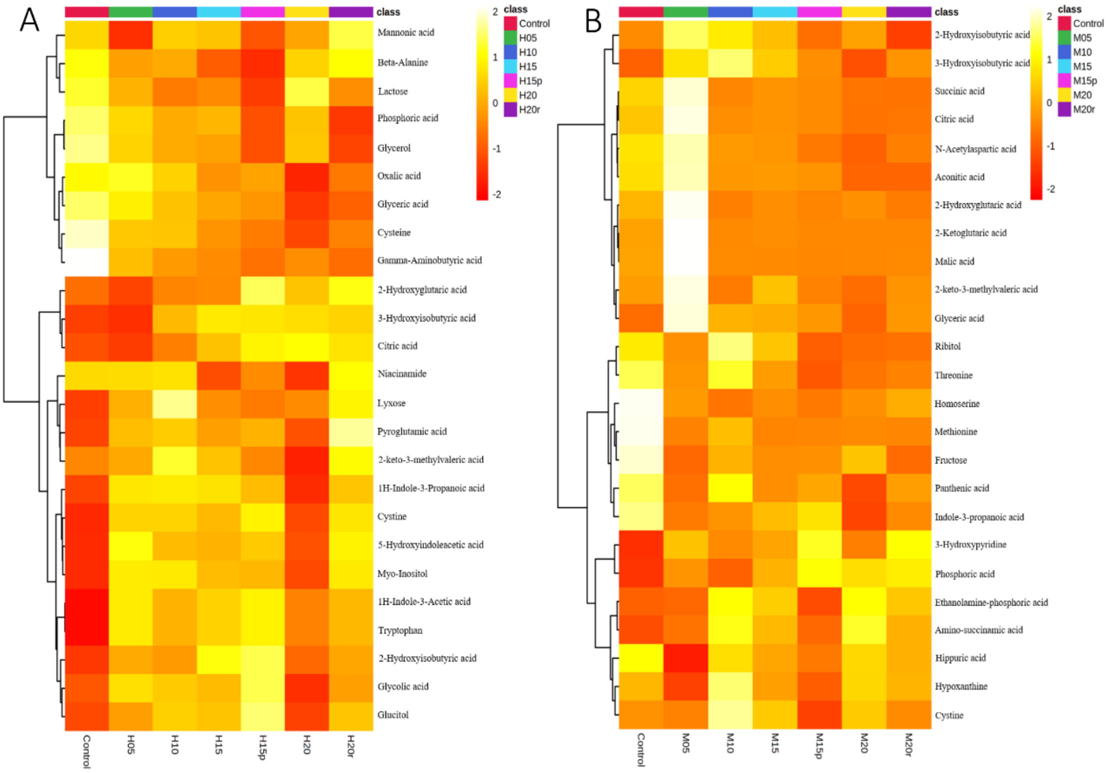

Note: Each row corresponds to a specific metabolite; each column corresponds to a set of samples (only group averages are shown). All the values were logarithmically transformed and are expressed as normalized values of the detected abundance of each metabolite. Red to orange indicates high to intermediate relative abundance, whereas orange to yellow indicates intermediate to low relative abundance.

Figure S8. The effects of morphine on metabolites in urine,.

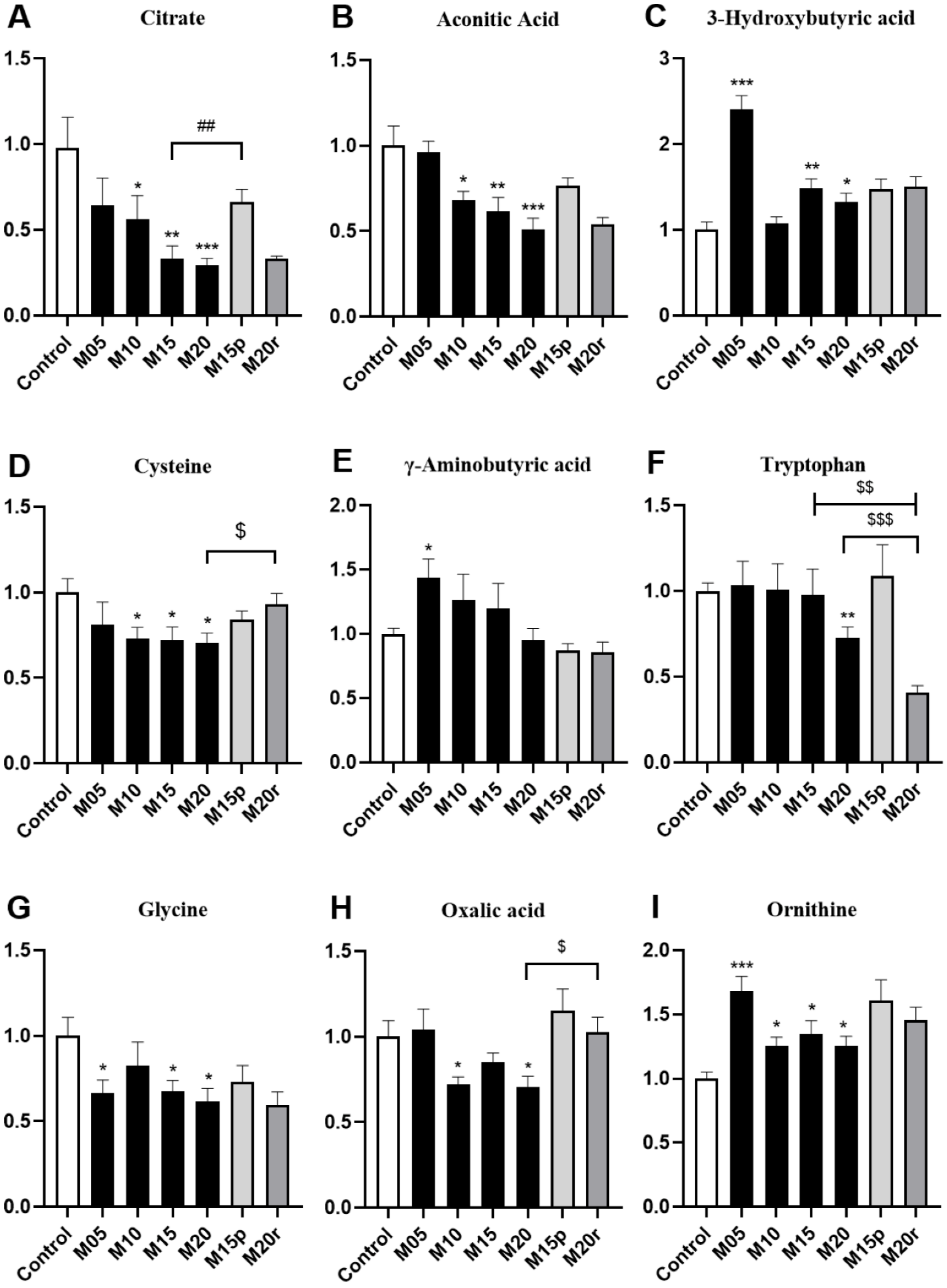

Note: H5, exposure to morphine for 5 days; H10, exposure to morphine for 10 days; H15, exposure to morphine for 15 days; H20, exposure to morphine for 20 days; H15p, withdrawal for 5 days after 10 days of morphine administration; H20r, relapsing morphine use for 5 days (*p < 0.05, **p < 0.01 and ***p < 0.001 compared with the control groups; H15p compared with H15, #p < 0.05, ##p < 0.01 and ###p < 0.001; H20r compared with H15 or H20, $p < 0.05, $$p < 0.01 and $$$p < 0.001).

Table S1. Overview of the changes of urinary metabolites about heroin and morphine,.

Metabolic pathways	Compounds	Heroin Urinary metabolites			Morphine Urinary metabolites		
		exposure	withdrawal	relapse	exposure	withdrawal	relapse
**Lipid metabolism**	Palmitic acid	↓↓	↑	-	↑↑	-	-
	Octadecanoic acid	↓↓	↑	-	↑↑	-	-
	3-Hydroxybutyric acid	↓↓	-	↓	↑↑	-	-
Carbohydrate metabolism	Citric acid	↑↑	-	↓	↓↓	↑↑	-
	Aconitic acid	↓↓	↓	-	↓↓	↑	-
	Lactic acid	↓↓	-	-	-	↑↑	↑↑
	Ribitol	↓↓	-	-	↓↓	↓	↓
GSH	Methionine	↓↓	↑	↑	↓↓	-	-
	Cysteine	↓↓	↓	-	↓↓	↑	↑
	Serine	↓↓	↓	↓	↓↓	-	↓
Amino acid metabolism	Aspartic acid	↓↓	-	-	↓↓	↓	↓
	Proline	↓↓	-	↓	↓	↓	↓
	Valine	↓↓	-	↑	↓↓	-	↓
	Threonine	↓↓	-	-	↓↓	-	-
	Glycine	↓↓	↓	↓	↓↓	↑	↓
	Tryptophan	↑↑	↑	-	-	↑	↓↓
	Leucine	↓↓	-	-	↓↓	-	↓
	Ornithine	↓↓	↑	-	↑↑	↑	-
Others	Oxalic acid	↓↓	↑	-	↓	↑	↑
	γ-Aminobutyric acid	↓↓	-	-	↑	↓	↓
	5-Hydroxytryptamine	↓↓	↓	↑	↑	↓↓	↓

Note: the sign indicates the direction of change; ↓↓ for decrease, ↑↑ for increase, - for no change ($p < 0.05$, as indicated by the statistical analysis T-test); ↓ for decrease, ↑ for increase (there was a trend, but it was not statistically significant). Comparing the metabolites changes from the control group with the continuous heroin or morphine exposure group(15 days), and both the withdrawal group and the relapse group are compared with the exposure group for 15 days.

Table S2. Overview of the changes of serum and urinary metabolites about heroin,.

Metabolic pathways	Compounds	Heroin Serum metabolites			Heroin Urinary metabolites		
		exposure	withdrawal	relapse	exposure	withdrawal	relapse
**Lipid metabolism**	Palmitic acid	↑↑	↑↑	↓↓	↓↓	↑	-
	Octadecanoic acid	↑↑	↓	-	↓↓	↑	-
	3-Hydroxybutyric acid	↑↑	↑	↓	↓↓	-	↓
Carbohydrate metabolism	Citric acid	↑↑	-	↓↓	↑↑	-	↓
	Lactic acid	↓↓	-	↓↓	↓↓	-	-
	Ribitol	↓↓	↓↓	-	↓↓	-	-
GSH	Methionine	↓	-	-	↓↓	↑	↑
	Cysteine	↓	-	↓	↓↓	↓	-
	Serine	↓↓	↑↑	↓	↓↓	↓	↓
Amino acid metabolism	Aspartic acid	↑↑	↓↓	↓↓	↓↓	-	-
	Proline	↓↓	-	↓↓	↓↓	-	↓
	Valine	↑↑	↓↓	-	↓↓	-	↑
	Threonine	↓↓	↑	-	↓↓	-	-
	Tryptophan	↓↓	↓	↓	↑↑	↑	-
	Leucine	↑↑	↓↓	-	↓↓	-	-

	Ornithine	-	-	-	↓ ↓	↑	-
Others	5-Hydroxytryptamine	↓	↑	-	↓ ↓	↓	↑
	Xanthosine	↑ ↑	-	-	↓ ↓	-	-

Note: the sign indicates the direction of change; ↓ ↓ for decrease, ↑ ↑ for increase, - for no change ($p < 0.05$, as indicated by the statistical analysis T-test); ↓ for decrease, ↑ for increase (there was a trend, but it was not statistically significant). Comparing the metabolites changes from the control group with the continuous heroin or morphine exposure group(15 days), and both the withdrawal group and the relapse group are compared with the exposure group for 15 days.

Table S3. Overview of the changes of serum and urinary metabolites about morphine.

Metabolic pathways	Compounds	Morphine Serum metabolites			Morphine Urinary metabolites		
		exposure	withdrawal	relapse	exposure	withdrawal	relapse
Lipid metabolism	Palmitic acid	↑	-	-	↑ ↑	-	-
	Octadecanoic acid	↑ ↑	↓	-	↑ ↑	-	-
	3-Hydroxybutyrate	↑	↑ ↑	↑	↑ ↑	-	-
Carbohydrate metabolism	Citrate	-	↓	↓	↓ ↓	↑ ↑	-
	Aconitate	↑ ↑	↓ ↓	↓ ↓	↓ ↓	↑	-
	Lactate	-	-	-	-	↑ ↑	↑ ↑
	Ribitol	↓ ↓	↑ ↑	-	↓ ↓	↓	↓
GSH	Methionine	↓ ↓	↑	↑ ↑	↓ ↓	-	-
	Cysteine	↓ ↓	↑ ↑	↑ ↑	↓ ↓	↑	↑
	Serine	↓ ↓	↑ ↑	↑ ↑	↓ ↓	-	↓
Amino acid metabolism	Aspartate	↓	↓	-	↓ ↓	↓	↓
	Proline	↓ ↓	↑	↑ ↑	↓	↓	↓
	Valine	↓	↓ ↓	↓	↓ ↓	-	↓
	Threonine	↓ ↓	↑ ↑	↑ ↑	↓ ↓	-	-
	Tryptophan	↓ ↓	↓	↓	-	↑	↓ ↓
	Leucine	-	↓	↓	↓ ↓	-	↓
	Ornithine	↓ ↓	↑ ↑	-	↑ ↑	↑	-
Others	γ-Aminobutyric acid	↓	-	↓	↑	↓	↓
	5-Hydroxytryptamine	↓ ↓	↑ ↑	↑	↑	↓ ↓	↓

Note: the sign indicates the direction of change; ↓ ↓ for decrease, ↑ ↑ for increase, - for no change ($p < 0.05$, as indicated by the statistical analysis T-test); ↓ for decrease, ↑ for increase (there was a trend, but it was not statistically significant). Comparing the metabolites changes from the control group with the continuous heroin or morphine exposure group(15 days), and both the withdrawal group and the relapse group are compared with the exposure group for 15 days.
